# A Fundamental Micro Scale Study of the Roles of Associated Gas Content and Different Classes of Hydrocarbons on the Dominant Oil Recovery Mechanism by CWI

**DOI:** 10.1038/s41598-019-42226-6

**Published:** 2019-04-12

**Authors:** Mojtaba Seyyedi, Pedram Mahzari, Mehran Sohrabi

**Affiliations:** 1 Centre for Enhanced Oil Recovery and CO2 solutions, Heriot-Watt University, Edinburgh, United Kingdom; 2 CSIRO Energy, Australian Resources Research Centre (ARRC), 26 Dick Perry Avenue, Kensington, WA 6151 Australia; 3 Department of Earth Sciences, University College London, London, United Kingdom

## Abstract

Various studies demonstrated new gaseous phase formation and oil swelling and viscosity reduction are the oil recovery mechanisms by carbonated water injection (CWI) with new gaseous phase formation being the major recovery mechanism for live oil systems. However, none of the previous studies investigated the influences of dissolved gas content of the oil and oil composition, on the new gaseous phase. This study attempts to provide insights on this area. Based on the results, during CWI as CO2 partitions into the oil the dissolved gas of the oil liberates, which leads to in-situ new gaseous phase formation. The dissolved gas content of the crude oil has a direct impact on the saturation and growth rate of the new gaseous phase. The new gaseous phase doesn’t form for oils that have an infinite capacity for dissolving CO2, such as light pure hydrocarbon components. Oils with limited capacity for dissolving CO2, such as heavy hydrocarbon components, are responsible for the formation of the new gaseous phase. Therefore for a live crude oil, the relatively heavier fractions of oil are responsible for triggering of the new gaseous phase and light to intermediate oil components control the further growth of the new gaseous phase.

## Introduction

Carbonated (CO_2_-enriched) water injection (CWI) is a water-based enhanced oil recovery (EOR) scenario in which water as the carrier fluid displaces dissolved CO_2_ in the porous medium^1^. Since in this EOR technique CO_2_ is dissolved in the injected brine, CO_2_ does not present in the form of free gas phase which would eliminate the risk of buoyancy-driven leakage as in the case of CO_2_ injection^2^. Furthermore, since CW-oil system has a more favorable mobility ratio than CO_2_-oil system, CO_2_ is more evenly distributed in the reservoir during CWI than CO_2_ injection^1^.

Several researchers^1,3–20^ showed the promising oil recovery potential of CWI. In their studies the obtained additional oil recovery by CWI was mainly attributed to two major mechanisms, which are oil swelling^1,3–20^ and oil viscosity reduction^1,3,5–7,9–12,14–20^. Their results^1,3–20^ showed that as carbonated water (CW) flows in the porous medium, it comes in contact with the resident oil where CO_2_ partitions into the oleic phase. This CO_2_ mass transfer leads to oil swelling^1,3–20^ and oil viscosity reduction^1,3,5–7,9–12,14–20^ and thereby, oil displacement, formation of an oil bank and additional oil recovery^1,3–20^. However, model oil (n-Decane)^1,3,8–10,13,15,16^, mineral oils^5,9,10,16,21,22^ and dead crude oils^1,4,6,7,11,12,14,17–20^ were used in the majority of previous works reported in the area of CWI. As a result, the role of the presence of dissolved gas in the live reservoir crude oil on the oil recovery mechanisms of CWI was not investigated by the previous works^1,3–22^.

For the first time, through an extensive series of high-pressure and high-temperature coreflood^23–25^, PVT^26^, micromodel^23,27,28^ and slim tube^26^ experiments, as well as using fully CH_4_-saturated crude oils (live crude oils), we revealed the positive impact of the presence of associated gas in crude oil on the performance and oil recovery mechanisms of CWI. According to our published micromodel experiments^23,27,28^, CO_2_ mass transfer from carbonated water into a fully-CH_4_ saturated crude oil (live crude oil) led to the formation and growth of a gaseous phase inside the oil phase. This phenomenon was not observed for the carbonated water-dead oil system^24^. The results of our PVT experiments^26^ performed for a system of CW and a fully CH_4_-saturated crude oil to a limited extent revealed the characteristics of the new gaseous phase. We found that as soon as CW front comes in contact with the fully CH_4_-saturated crude oil and CO_2_ partitions into the live oil, the CO_2_ pushes the CH_4_ out of the solution, which leads to the formation of the new gaseous phase^26^. As CO_2_ partitioning into the oil phase continues, to keep the liquid-liquid-gas equilibrium, some portion of the dissolved CO_2_ in the oil phase transfers into the new gaseous phase^26^. As the new gaseous phase becomes richer with CO_2_, CO_2_ extracts some hydrocarbon components of the crude oil which in turn leads to the enrichment and further growth of the new gaseous phase^26^. Also, it was demonstrated that the presence of intermediate components in the associated gas can affect the new gaseous phase formation, which may lead to *in-situ* miscibility between the new gaseous phase and oil^29^. According to our published pore scale studies^23,27,28^, the formation of the new gaseous phase yields a stronger oil recovery by CWI through: (i) causing strong overall oil swelling which causes reconnection and redistribution of trapped oil ganglions and oil displacement, (ii) creating a favourable three-phase flow region with less residual oil saturation, and (iii) restricting the flow path of CW and diverting CW toward unswept areas of the porous medium. Furthermore, based on our previous findings^23–26^, formation and growth of the new gaseous phase play the dominant role in enhancing oil recovery by CWI in live oil condition. So far, our previous findings indicated the need for having an in-depth understanding of the characteristic of the new gaseous phase and factors influencing it.

Until now, the impacts of two important factors, which are the associated gas content of the oil and the oil composition, on the formation and growth of the new gaseous phase have not been fully investigated and require a fundamental study at pore scale. Since most oil reservoirs have live crude oils with different associated gas contents and oil compositions^30^, studying the influences of the abovementioned factors on the new gaseous phase is of value. Therefore, gaining an in-depth understanding of these factors may improve the viability of this EOR scenario (i.e. CWI) in field applications. For the first time, through this study, we attempted to comprehensively investigate the impacts of these factors on the new gaseous phase. With this objective, a series of high-pressure and high-temperature direct-flow visualization (micromodel) experiments were designed and conducted. First, to study the role of associated gas content of reservoir crude oil on the liberation and growth of the new gaseous phase, a reservoir crude oil at different saturation conditions was used. Next, to address the role of different classes of hydrocarbons, live synthetic oils made by mixing pure hydrocarbon components, and live crude oils modified in hydrocarbon composition were employed. Dynamic fluid-fluid interactions, at micro-scales, were observed at a pressure and temperature of 2500 psi and 38 °C.

## Experimental Setup and Procedure

### Micromodel Rig

**Figure 1:**
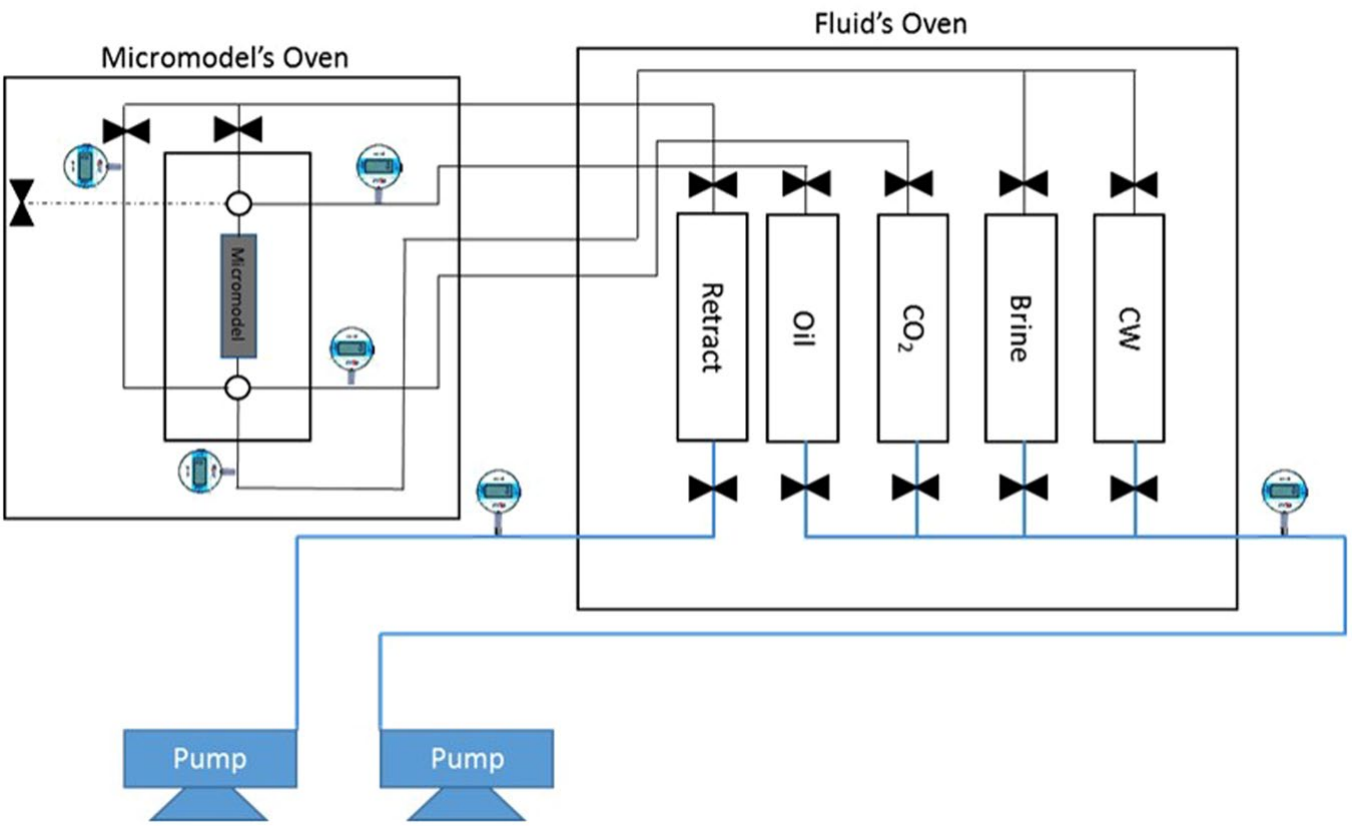
Schematic of high-pressure and high-temperature micromodel rig.

**Figure 2:**
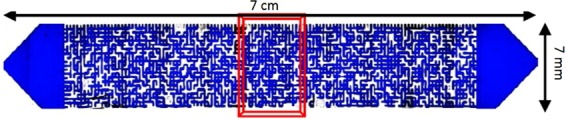
The micromodel when it is fully saturated with blue-dyed water.

Coreflooding is a common tool for investigating the oil recovery performance of any EOR strategy, including CWI^1,10–13,17,24,25^. The black box nature of coreflood experiments provides minimum information regarding the physics of process and oil recovery mechanisms by any EOR strategy, including CWI. To overcome this issue, direct visualization of fluid flows and interactions in the porous medium can be essential. In this study, a uniquely designed in-house micromodel rig, with the ability to work at pressures as high as 10,000 psi and temperatures as high as 100 °C, was employed. Figure 1 shows the schematic of the micromodel rig. All fluids were kept inside the fluid storage oven at test temperature and pressure. The micromodel was housed inside a separate oven under the same experimental condition. The micromodel was a transparent porous medium made of two sapphire glass plates which was placed inside a high-pressure chamber. The high-pressure chamber provided the confining pressure on the micromodel. The confining pressure was kept 400 psi higher than the pressure inside the micromodel at all times. One of the palates was etched with a pattern to allow fluids to flow in a controlled way and the other one was unetched to seal the system. The pattern was a heterogeneous pattern synthesized in-house to lead to high residual oil saturation after a waterflood. The average pore depth of the micromodel was 50 micrometers and the pore-throat diameters ranged from 300 to 500 micrometers. The depth of the pores was measured by a light scattering method. The micromodel dimensions are summarized in Table 1. The porosity and pore size of the micromodel was measured by image analysis when the micromodel was fully saturated with blue-dyed water (Fig. 2). The permeability of the micromodel was around 7 D.

**Table 1:** Micromodel dimensions.

length (cm)	width (cm)	pore volume (cm^3^)	porosity	permeability (D)	average pore depth (µm)	pore diameter range (µm)
7	0.7	0.01	0.5109	7	50	30–500

The glass walls of the micromodel provide the user with the direct observation of fluid flow and fluid-fluid interactions at the pore scale under high-pressure and high-temperature conditions. To capture high-quality images and videos at the micro scale during flooding stages, a high-resolution microscope kit was used. The microscope had a built-in fine focus. The kit was fixed at the desired position by utilizing a manual camera mount and positioning system. The camera was connected to a PC where Streampix software was used for recording videos and pictures. In this study, micromodel experiments were conducted at a pressure and temperature of 2500 psi and 38 °C.

### Fluids Properties

**Figure 3:**
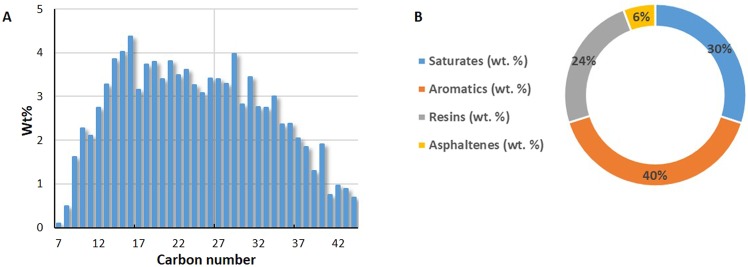
Crude J Hydrocarbon composition (**A**) and properties (**B**).

To address the effects of the associated gas content of the oil, and different classes of hydrocarbons on the new gaseous phase, various oil types were used. Table 2 shows the list of utilized oils. Crude J is a medium viscosity reservoir crude oil with the hydrocarbon composition and properties presented in Fig. 3A,B, respectively. The hydrocarbon composition of crude j was obtained by using a high-resolution gas chromatograph with a flame ionization detector (FID). The viscosity and API of crude J are 86 cP and 20.87. Pure hydrocarbons, such as C6, C10, C16, C17, and C24 were also used to investigate the roles of different classes of hydrocarbons on the new gaseous phase formation. Experiments 1 and 3 are similar to the experiments reported in the author’s previous publications^24,26–28^. These experiments in combination with experiment 2 can provide insights into the role of the associated gas content of crude oil on the fluid-fluid interactions occurring during carbonated brine injection. This was the reason for re-performing and reporting experiments 1 and 3 in this study.

**Table 2:** Utilized oils.

experiment number	oil used in the experiment	purpose
1	Dead crude J	
2	Half CH_4_-saturated crude J	
3	Fully CH_4_-saturated crude J	
4	Fully CH_4_-saturated C6	
5	Fully CH_4_-saturated C10	
6	Fully CH_4_-saturated mixture of 30 wt% crude J + 70 wt% C10	
7	Fully CH_4_-saturated mixture of 50 wt% crude J + 50 wt% C10	
8	Fully CH_4_-saturated C16	
9	Fully CH_4_-saturated C17	
10	Fully CH_4_-saturated mixture of 77 wt% C17 + 23 wt% C24

To saturate the oil types used in this study with CH_4_, the oil was mixed with CH_4_ at 2450 psi and 38 °C. This 50 psi difference between the saturation pressure (2450 psi) of oils and the test pressure (2500 psi) would ensure no gas liberation due to pressure variations. To prepare CW, a synthetic sea brine, with the ionic composition presented in Table 3, was mixed with CO_2_ with a purity of 99.99 mole % at 2450 psi and 38 °C.

**Table 3:** Ionic composition of brine.

ion	ppm
Na^+^	16844
Ca^+2^	664
Mg^+2^	2279
SO_4_^−2^	3560
Cl^−^	31107
HCO_3_^−1^	193

### Methodology

Having prepared the fluids, they were transferred to the fluid storage oven. Initially, the micromodel was fully saturated with brine (Table 3). Next the desired oil at the rate of 0.1 cc/h, was injected into the micromodel to displace the water and establish the initial water saturation. The injection of oil was continued for several pore volumes until no further changes in the fluids saturation and distribution was detected. Having established the initial water and oil saturation, to study the dynamic fluid-fluid interactions that happen during CWI, the model was flooded with CW. The injection rate was 0.01 cc/h and the direction of injection was from the bottom of the micromodel toward its top. The injection of CW was continued for around 24 hours. During each test, the micromodel was scanned by the high-resolution microscope kit to detect any possible fluid-fluid interactions at pore (micro) scale. For further details about the experimental procedure, the reader is referred to the author’s previous publications^27,28^.

## Results and Discussion

The main objective of this study is to investigate the dynamic phase behavior between CW and different oil types with the main focus being on the new gaseous phase. Since factors such as different oil viscosities could affect fluids flow in the porous medium when different oils were used, fluid displacement and oil recoveries could not be comparatively investigated in this work.

### Roles of Associate Gas Content of Crude Oil

**Figure 4:**
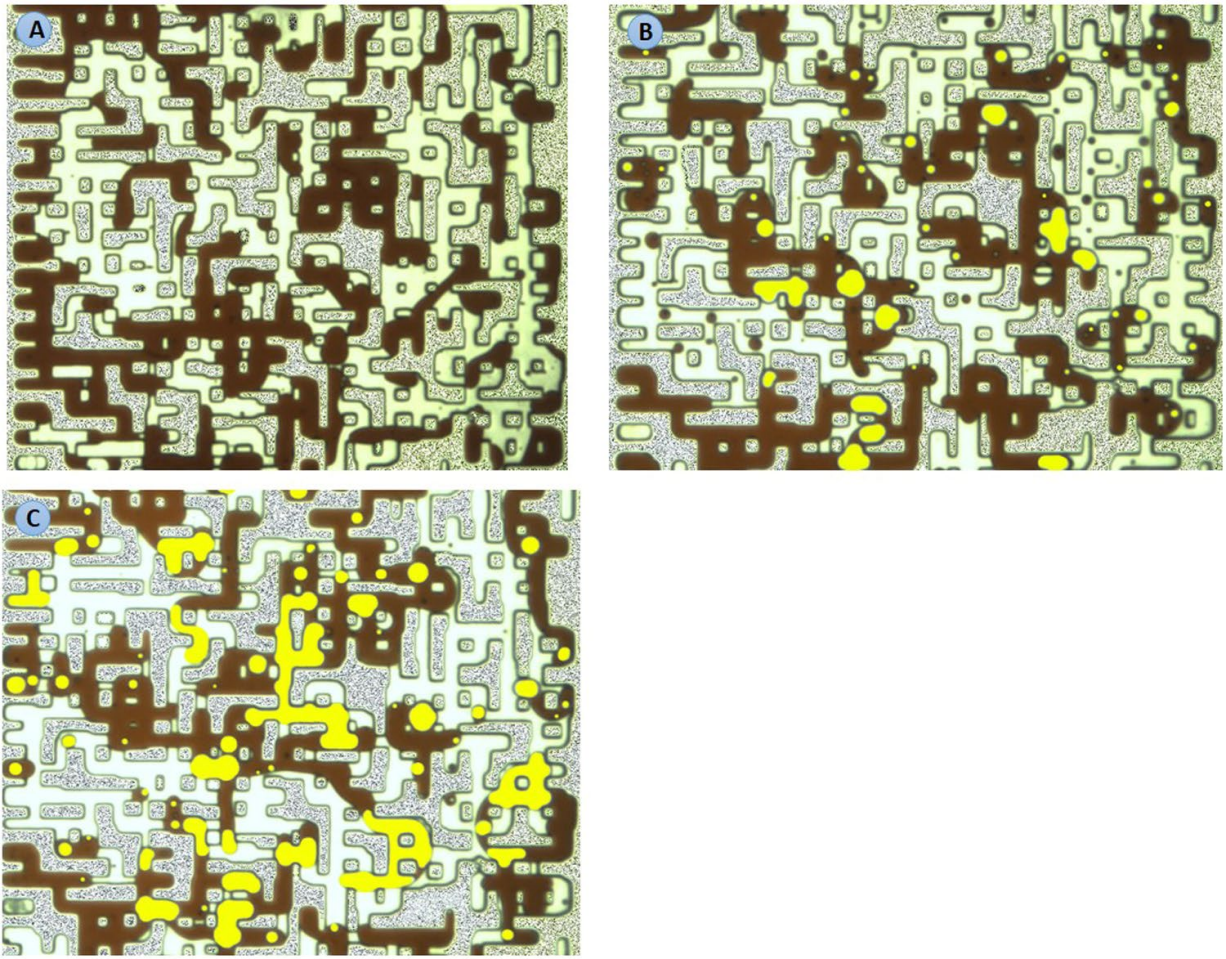
A magnified section of the micromodel after 10 hours of CWI in (**A**) dead crude oil, (**B**) half CH_4_-saturated crude oil, and (**C**) fully CH_4_-saturated crude oil systems. The brown, white and yellow areas indicate the oil, CW and the new gaseous phase, respectively.

The purpose of experiments 1 to 3 was to investigate the impacts of the associated gas content of crude oil on the new gaseous phase. Dead crude oil, partially (half) and fully-CH_4_ saturated crude oils were used in these tests. Figure 4A–C show a magnified section of the micromodel after 10 hours of CWI in these three systems. As observed in Fig. 4A, the new gaseous phase did not form during CWI in the dead oil system. Conversely, during CWI in partially CH_4_-saturated crude J system (Fig. 4B), after around 1 hour of CWI, small bubbles of a new gaseous phase formed inside the oil phase and as the injection continued, their saturation increased. The yellow areas in Fig. 4B and other figures of this study highlight the new gaseous phase formed during CWI. These results indicate that the presence of associated gas in crude oil is vital for the new gaseous phase formation.

Figure 4C shows the same section of micromodel after 10 hours of CWI in a system where crude J was fully saturated with CH_4_. In this system, as soon as CW breakthrough happened, the new gaseous phase formed in the oil phase and as CWI continued the saturation of the new gaseous phase increased. Comparison of the results of partially (half) and fully CH_4_-saturated crude J indicated the positive impact of associated gas content of crude oil on the formation and growth of the new gaseous phase. Formation and growth of the new gaseous phase occurs faster and stronger for a given crude oil type when its associated gas content is higher.

### Role of Oil Composition

The purpose of experiments 4 to 10 was to address the role of different classes of hydrocarbons on the new gaseous phase. In this study, oil components were divided into two categories; i) oil components (i.e. C6 and C10) that are miscible, like first contact miscible (FCM) processes, with CO_2_ at test conditions, and ii) heavy oil components (such as C16 and C17) which partially dissolve CO_2_.

#### Role of First Contact Miscible (FCM) Oil Components

**Figure 5:**
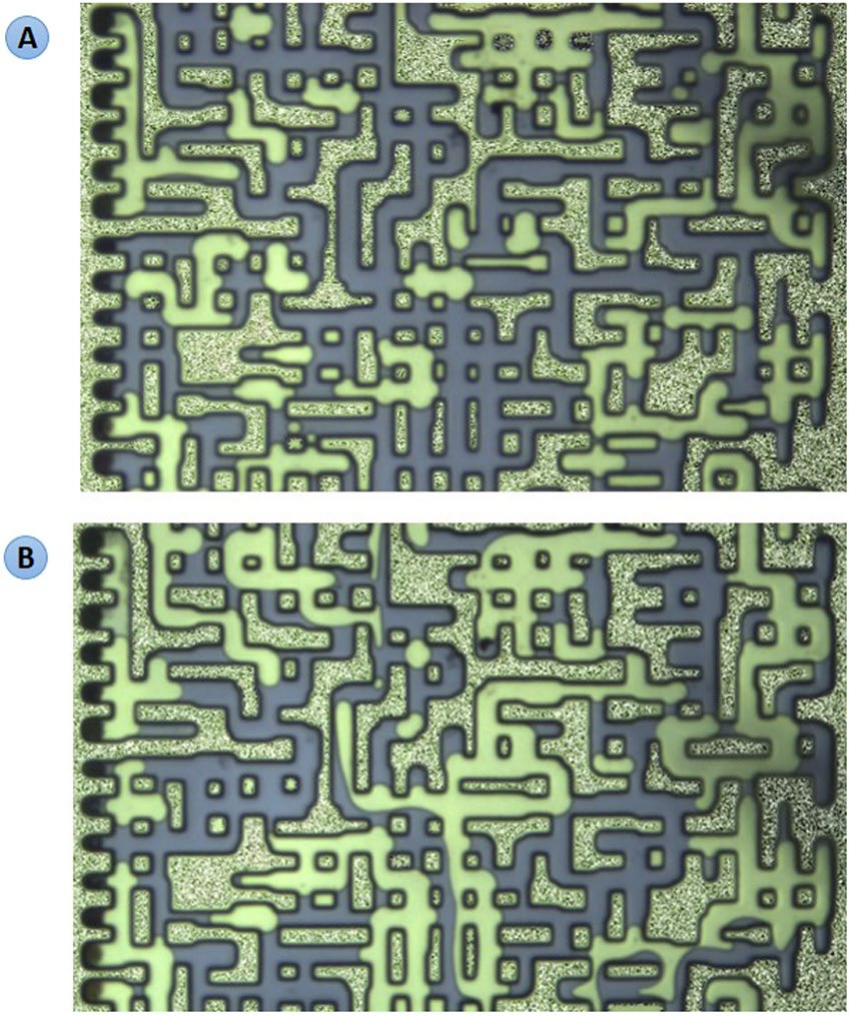
CO_2_ partitioning between CW (in purple color) and (**A**) CH_4_-saturated C6 (in white color) and (**B**) CH_4_-saturated C10 (in white color) led to normal oil swelling. No indication of the new gaseous phase formation was observed.

To address the influence of FCM oil components on the new gaseous phase, two different hydrocarbon components (C6 and C10), which are FCM with CO_2_ under the experimental condition, were used. Figure 5 shows a magnified section of the micromodel after 24 hours of CWI for two different systems including CH_4_-saturated C6 (Fig. 5A) and CH_4_-saturated C10 (Fig. 5B). During the long period of carbonated water injection (more than 40 hours) in these systems, the new gaseous phase did not form. The only mechanism that improved the oil recovery was normal oil swelling which in turn led to oil reconnection and redistribution and therefore further oil recovery.

**Figure 6:**
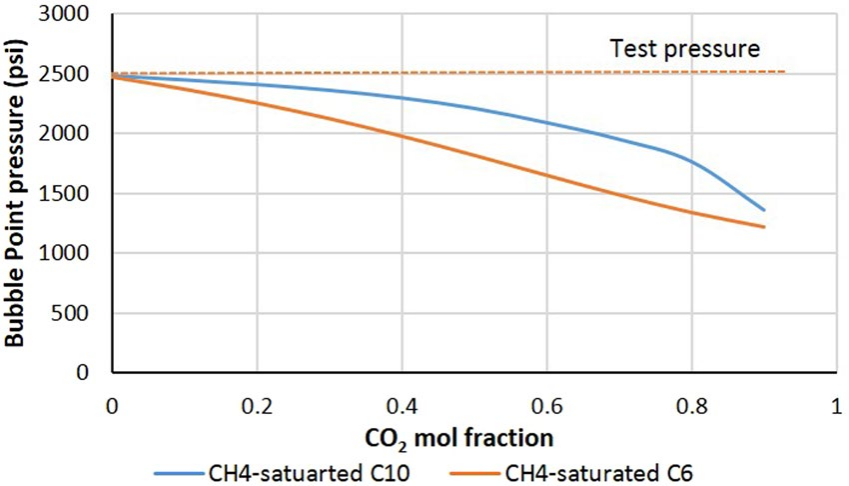
Bubble point pressure of CH_4_-saturated C6/C10 at different CO_2_ mole fractions.

By using the Peng Robinson equation of states built in the PVT package of CMG software, the ternary system of CO_2_, CH_4_, and C6/C10 was analyzed. Since the system under study is composed of pure components with known EOS parameters, the results of EOS analysis for these ternary systems are expected to be close to laboratory experiments^31^. In the built model, the live oil was loaded into a cell and the temperature was set at 38 °C. The bubble point of the live oil was measured. Then, a small amount of CO_2_ was transferred into the cell and a new saturation pressure was identified. This process was repeated for different CO_2_ mole fractions. Figure 6 shows the bubble point pressure of CH_4_-saturated C6/C10 versus CO_2_ concentration. As the CO_2_ mole fraction increased, the bubble points of live C6 and C10 decreased and always remained under the operating pressure of our tests (red line). Therefore, both systems remained in the liquid phase. These trends shows that CO_2_ dissolution in CH_4_-saturated C6 and C10 do not lead to new gaseous formation which shows the unlimited solubility of CO_2_ in these systems. The unlimited solubility of CO_2_ in these systems indicates first contact miscibility between CO_2_ and these live model oils.

Consistent with modeling results, micromodel experiments also showed that FCM oil components have an infinite capacity for dissolving CO_2_ in themselves while keeping CH_4_ in solution (Fig. 5). Therefore, CO_2_ mass transfer from CW into these components does not have the potential to push the dissolved CH_4_ out of the solution and trigger the new gaseous phase formation. In other words, CO_2_ and CH_4_ dissolved in FCM hydrocarbon components do not compete for remaining in solution (oil phase). As a result, FCM oil components are not responsible for triggering the formation of the new gaseous phase during CWI in live oil systems. Furthermore, it can be concluded that the new gaseous phase would not be formed for those oil types that are FCM with CO_2_. This conclusion is regardless of the associated gas content of these oil types. However, in real oil reservoirs, reservoir oils are seldom FCM with CO_2_; therefore, the new gaseous phase would be formed during CWI in oil fields where oil has associated gases.

**Figure 7:**
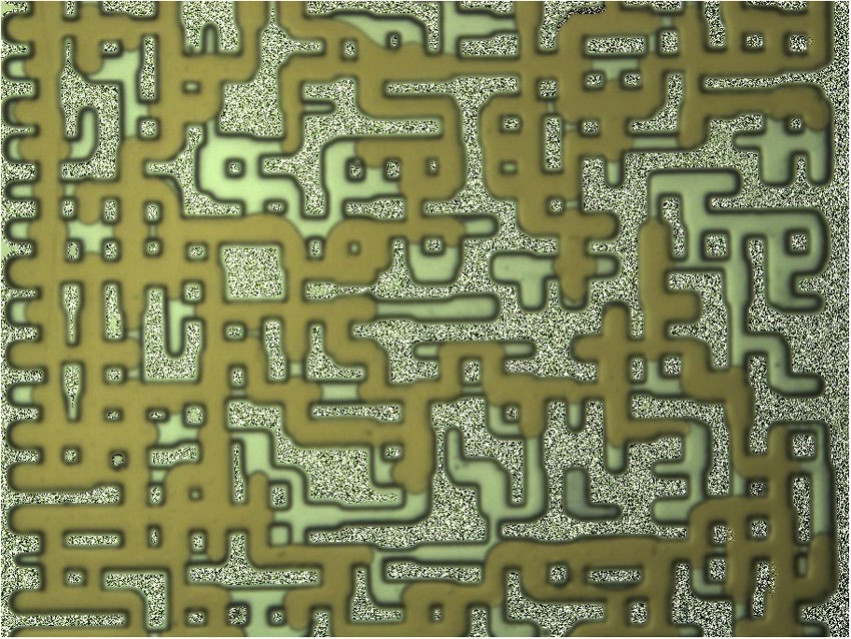
A magnified section of the model after establishing initial water (in white color) and oil (30 wt% crude J + 70 wt% C10) saturation. The oil is shown in brown color.

To further study the role of FCM hydrocarbon components in a crude oil on the new gaseous phase, two different mixtures of crude J and C10 were prepared. The first mixture was made of 30 wt% crude J and 70 wt% C10, and the second mixture was made of 50 wt% crude J and 50 wt% C10. Both mixtures were fully saturated with CH_4_. The modified crude oils were not FCM with CO_2_. Figure 7 shows a section of the micromodel after establishing initial water and oil (30 wt% crude J + 70 wt% C10) saturation.

**Figure 8:**
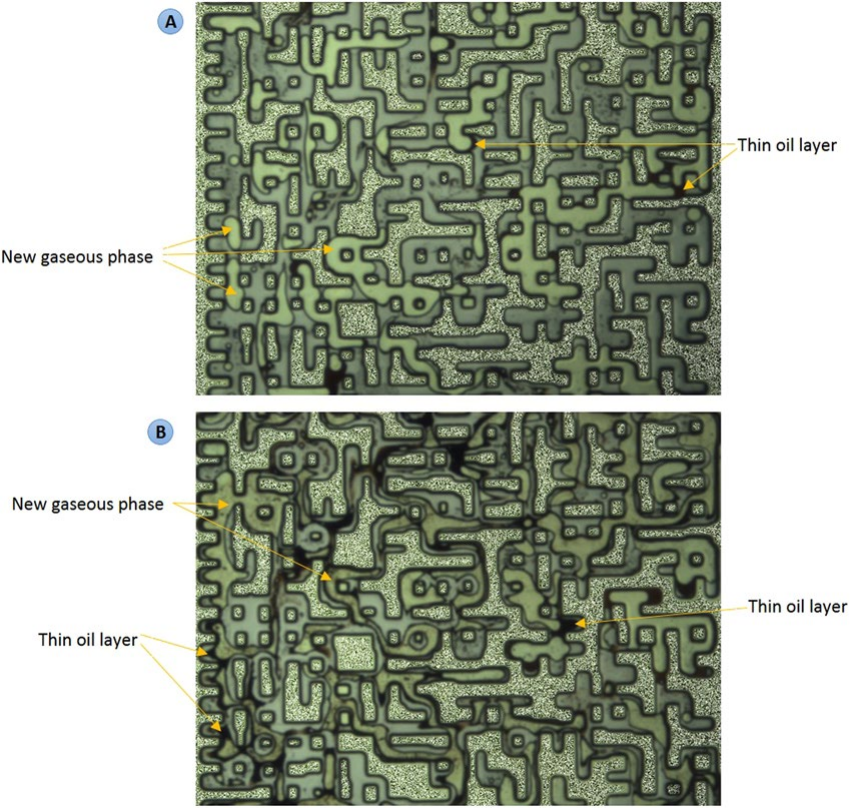
A magnified section of the micromodel after 10 h of CWI (shown in purple color) in the CH_4_-saturated mixture of (**A**) 30 wt% crude J + 70 wt% C10 and (**B**) 50 wt% crude J + 50 wt% C10. The new gaseous phase (in white color) is surrounded by thin black layers of oil.

The results of CWI for both modified oils were compared with each other and with that of CH_4_-saturated crude J. Fig. 8 shows a magnified section of the micromodel after 10 hours of CWI in both modified oil systems. In both tests, a unique behavior was observed. A high saturation of the new gaseous phase was surrounded by a thin black layer of oil. During both tests, the majority of the oil phase was extracted into the new gaseous phase leading to a significant overall oil swelling. For these modified oils, as soon as CW came in contact with the oil phase, a rapid formation of gas bubbles in the oleic phase was observed following by a strong extraction of the oil components into the new gaseous phase. This extraction enriched the new gaseous phase. In the micromodel, under controlled experimental condition, a change in the oil quality can be detected based on the change in the oil color. The oil that is under extraction has a darker color compared to the original oil^27^. Comparison of the color of original oil (Fig. 7) with that of CW-contacted oil (Fig. 8A) reveals the presence of this strong extraction. This behavior is due to the enrichment of the new gaseous phase with CO_2_^26^_,_ which in turn leads to extraction of light to intermediate oil components into the new gaseous phase and its further enrichment. The observed strong extraction from the oleic phase into the new gaseous phase significantly increased the saturation of the new gaseous phase. The saturation of the new gaseous phase during CWI in the mixture of 30 wt% crude J + 70 wt% C10 (Fig. 8A) was higher than the mixture of 50 wt% crude J + 50 wt% C10 (Fig. 8B). This indicates that the new gaseous phase was mostly made of the extracted C10.

Table 4 presents the amounts of overall oil swelling for a trapped oil ganglion after 12 hours of CWI in experiments 3 and 6. Overall oil swelling is defined as:

$$\frac{{\rm{Final}}\,{\rm{volume}}\,{\rm{of}}\,{\rm{the}}\,{\rm{hydrocarbon}}\,\mathrm{phase}\,({\rm{oil}}+{\rm{new}}\,{\rm{phase}})-{\rm{Initial}}\,{\rm{volume}}\,{\rm{of}}\,{\rm{the}}\,{\rm{oil}}}{{\rm{Initial}}\,{\rm{volume}}\,{\rm{of}}\,{\rm{the}}\,{\rm{oil}}}\times 100$$

**Table 4:** Overall oil swelling during CWI in experiments 3 and 6.

Utilized oil	Overall oil swelling
CH_4_-saturated Crude J	35%
Mixture of 70 wt% C10 + 30 wt% Crude J	150%

The strong extraction of C10 from modified crude oils into the new gaseous phase led to a significantly stronger overall oil swelling compared to the live crude J. This can positively influence the extra oil recovery that can be obtained by CWI.

**Figure 9:**
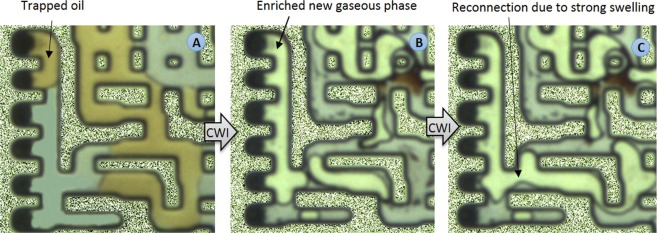
Trapped oil (mixture of 70 wt% C10 + 30 wt% crude J) ganglion (**A**) at the initial time and (**B**,**C**) at the end of CWI.

Figure 9 shows the swelling of a trapped oil made of 70 wt% C10 and 30 wt% crude J during CWI. The reasons for such a significant high overall oil swelling were the dynamic transfer of CO_2_ into the new gaseous phase and more importantly the strong extraction of the light to intermediate oil components into the new gaseous phase. This strong swelling led to the reconnection of the trapped oil to other bypassed hydrocarbon phases and oil displacement.

**Figure 10:**
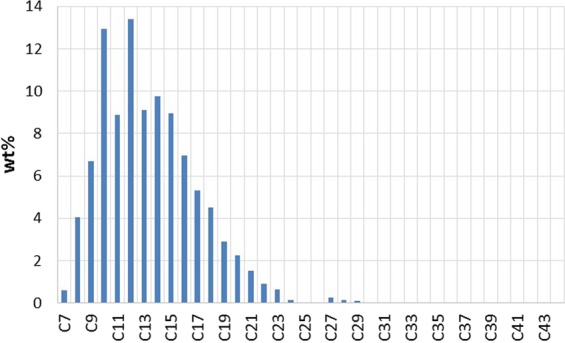
Composition of the condensate formed after an extensive cell volume of CW brought in contact with live crude J^26^.

Based on the results of this section, although FCM oil components in live reservoir crude oils are not responsible for triggering the new gaseous phase, they can lead to further growth of the new gaseous phase. As the new gaseous phase is getting enriched with CO_2_, FCM oil components (light to intermediate hydrocarbon components) can be extracted into the new gaseous phase leading to further growth of the new gaseous phase which in turn leads to a stronger overall oil swelling, oil reconnection and redistribution, and finally further oil recovery. This extraction was reported by micromodel tests where extraction of light components (C4 and C3) led to *in-situ* miscibility between the oil and the new gaseous phase^29^. The presence of this extraction was also confirmed by our PVT experiments^26^ in which after a high volume of CW contacted live crude J, the formation of a small volume of the condensate was observed. Figure 10 shows the condensate composition. It should be noted that the composition of condensate is a direct function of the oil composition. The condensate formed during CW-crude J PVT tests is mainly made of light to intermediate oil components, in particular C10 and C12.

**Figure 11:**
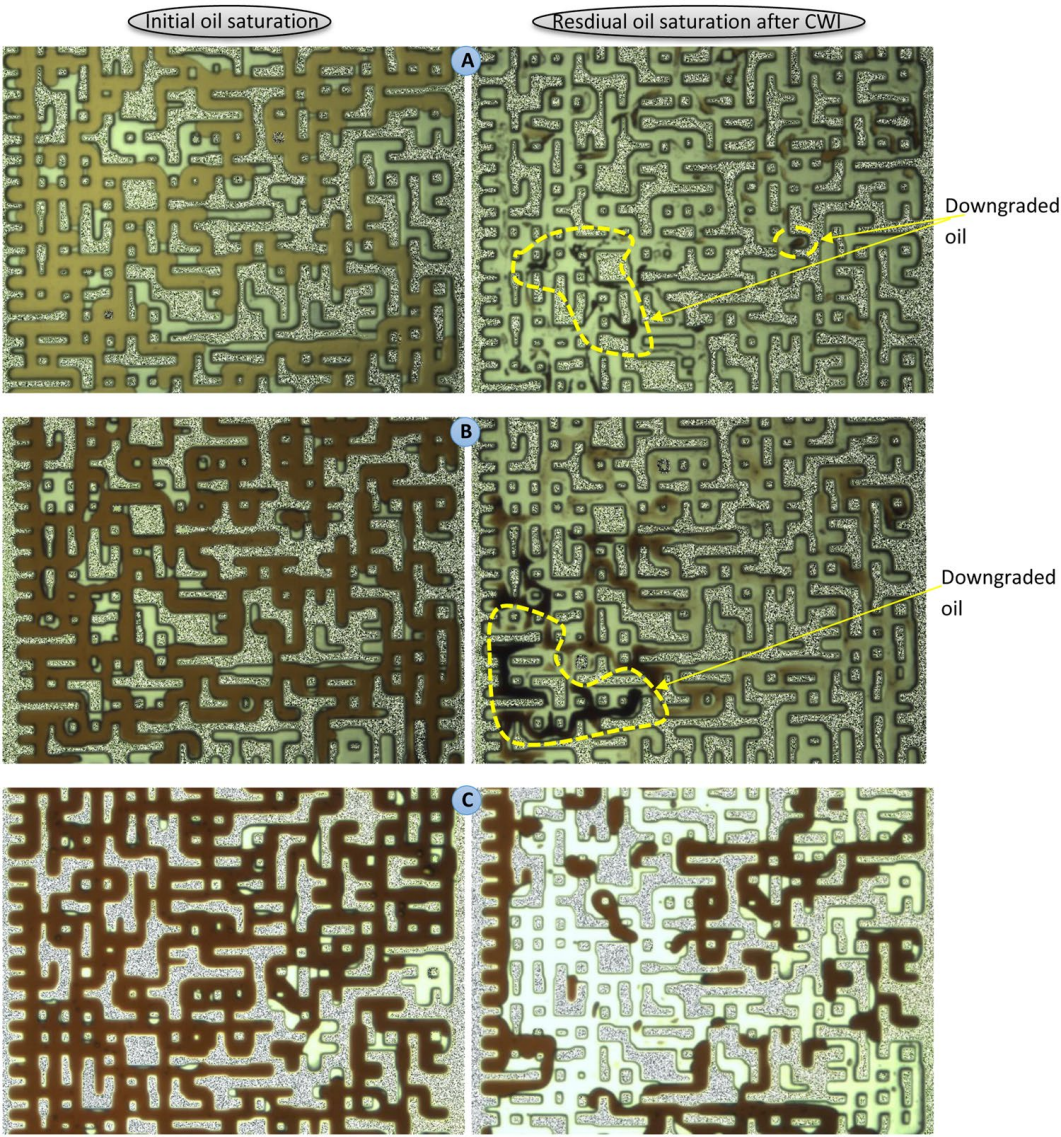
Comparison of the oil compositional variations and oil recovery potentials during CWI in three different systems, including (**A**) CH_4_-saturated mixture of 30 wt% crude J + 70 wt% C10, (**B**) CH_4_-saturated mixture of 50 wt% crude J + 50 wt% C10, and (**C**) CH_4_-saturated crude J.

Although the extraction of FCM oil components from the oil phase into the new gaseous phase helps to further its growth and thereby improve oil recovery during CWI, this extraction has an adverse compositional effect over the quality of the remained oil. To investigate the impact of this extraction on the quality of the remained oil after CWI, the micromodel was flooded with sea brine (Table 3). Through this final waterflooding step, the dissolved CO_2_ was stripped out of the remaining oil and the actual remaining oil volume and color were detected. The rate of brine injection was 0.01 cc/h and brine was injected from the same head as CW was injected. During this step, no oil displacement was observed and only the remaining oil ganglia were shrunken as their CO_2_ was extracted. Figure 11 compares the color of the remaining oil after CWI in experiments 3, 6 and 7, with their original oil color. In experiments 6 and 7, where C10 content of crude oil was enhanced, the formation and growth of the new gaseous phase led to a very strong oil recovery, while at the same time leading to an adverse oil compositional effect. Conversely, for fully CH_4_-saturated crude J, formation and growth of the new gaseous phase did not cause any detectable negative effect on the oil composition and the remaining oil looked similar to its initial condition. This shows that the negative impact of extraction of light hydrocarbon components into the new gaseous phase is a direct function of the light components content of the crude oil. For crude oils with higher amounts of light hydrocarbon components, during CWI the extraction to the new gaseous phase will be stronger, and thereby oil quality would be more affected.

#### Role of Heavy Oil Components

**Figure 12:**
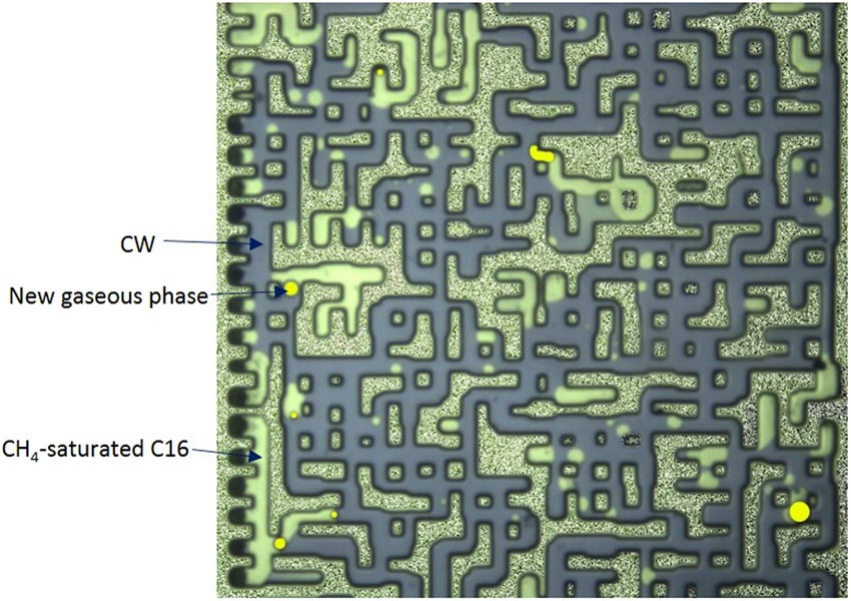
A magnified section of the micromodel after 10 hours of CWI for the system initially saturated with CH_4_-saturated C16. CO_2_ partitioning from CW (in purple color) into live C16 (in white color) led to the formation of the new gaseous phase (yellow areas).

**Figure 13:**
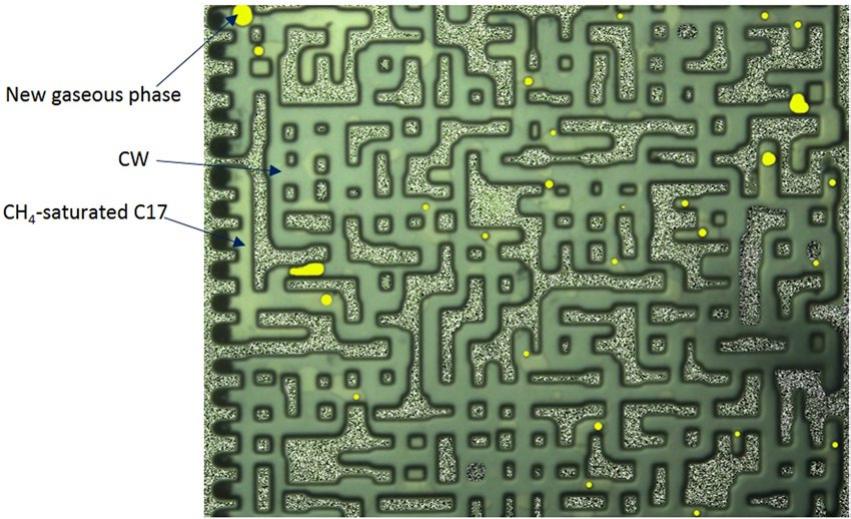
A magnified section of the micromodel after 10 hours of CWI for the system initially saturated with CH_4_-saturated C17. CO_2_ partitioning from CW into live C17 led to the formation of the new gaseous phase (yellow areas).

**Figure 14:**
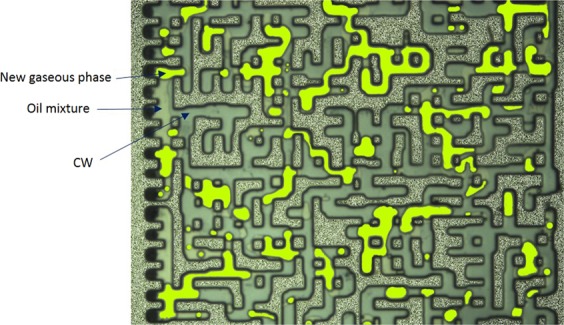
A magnified section of the micromodel after 10 hours of CWI for the system initially saturated with the CH_4_-saturated mixture of 77 wt% C17 and 23 wt% C24. CO_2_ partitioning into oleic phase led to the formation of the new gaseous phase (yellow areas).

To adequately investigate the impact of heavy oil components on the new gaseous phase during CWI, experiments 8 to 10 were carried out. Figures 12, 13 and 14 present a magnified section of the micromodel after 10 hours of CWI in the model saturated with C16, C17 and a mixture of 77 wt% C17 and 23 wt% C24, respectively. The employed oils were fully saturated with CH_4_ under the experimental conditions.

CWI in these systems led to the rapid formation of the new gaseous phase inside the oleic phase. Based on these observations, it can be concluded that heavy hydrocarbon components are in charge of triggering the formation of the new gaseous phase since they have a limited capacity for holding both CH_4_ and CO_2_ in their own. Therefore, a competition exists between CH_4_ and CO_2_ to remain in the solution (oil phase) which in turn leads to the formation of the new gaseous phase.

As seen from Figs 12, 13 and 14, formation and growth of the new gaseous phase for the case of live oil mixture (77 wt% C17 and 23 wt% C24) was stronger than live C17 and for live C17 was stronger than live C16. This behavior was observed while the associated gas content of the live oil mixture (77 wt% C17 and 23 wt% C24) was less than live C17 and the associated gas content of live C17 was less than live C16 under the experimental conditions. As a result, the associated gas content of the oil is not the only factor that controls the strong and fast formation of the new gaseous phase. Therefore, for two different live reservoir crude oils, the one with higher associated gas content does not necessarily have the stronger formation of the new gaseous phase during CWI. The impact of oil composition must be considered alongside its associated gas content.

Based on our observations, during CWI in the micromodel saturated with a CH_4_-saturated mixture of 77 wt% C17 and 23 wt% C24, the new gaseous phase formed ahead of the CW front in the porous media, while during CWI in the micromodel saturated with either CH_4_-saturated C17 or CH_4_-saturated C16, the new gaseous phase formed slightly after CW breakthrough. These observations revealed the role of each component on formation of the new gaseous phase. For the heavier oil components, the onset of new gaseous phase formation is sooner. This is attributed to the smaller capacity of heavier oil components for holding both CO_2_ and CH_4_ in solution compared to intermediate components. Consequently, it can be inferred that, during CWI, due to CO_2_ transfer from CW into the crude oil, the heavy oil components tend to release their dissolved CH_4_ which triggers the formation of the new gaseous phase.

## Conclusion

Based on the results of this study, it can be concluded that the presence of dissolved gas in oil is essential for the formation of the new gaseous phase during carbonated water injection. However, regardless of the dissolved gas content of the oil, if the oil is first contact miscible with CO_2_ at reservoir conditions such as light model oils, the new gaseous phase would not be formed. Light to intermediate hydrocarbon components of a crude oil facilitate further growth of the new gaseous phase as they can be extracted into the new gaseous phase while heavy hydrocarbon components are responsible for the formation of the new gaseous phase. The observed *in situ* new gaseous phase formation would be more profound in live crude oils, at or close to their bubble points, with a higher amount of first contact miscible hydrocarbon components, which can control the CO_2_ dissolution into the oil as well as the further growth of the new gaseous phase. Small quantities of heavy components would suffice for triggering the new gaseous phase formation and continuous feed of dissolved CO_2_ would lead to the favorable growth of the new gaseous phase. Therefore, as long as the injection of carbonated water continues and CO_2_ partitions from carbonated water into the trapped oil in the porous media, the saturation of the new gaseous phase inside the oil phase increases.

## Data Availability

All data generated or analyzed during this study are included in this published article.
